# Copper and cuproptosis: new therapeutic approaches for Alzheimer’s disease

**DOI:** 10.3389/fnagi.2023.1300405

**Published:** 2023-12-19

**Authors:** Xiao Li, Xinwang Chen, Xiyan Gao

**Affiliations:** ^1^Henan University of Chinese Medicine, Zhengzhou, Henan, China; ^2^College of Acupuncture-Moxibustion and Tuina, Henan University of Chinese Medicine, Zhengzhou, Henan, China; ^3^Acupuncture Clinic of the Third Affiliated Hospital of Henan University of Chinese Medicine, Zhengzhou, Henan, China

**Keywords:** copper, cuproptosis, Alzheimer’s disease, long-term potentiation, chelators

## Abstract

Copper (Cu) plays a crucial role as a trace element in various physiological processes in humans. Nonetheless, free copper ions accumulate in the brain over time, resulting in a range of pathological changes. Compelling evidence indicates that excessive free copper deposition contributes to cognitive decline in individuals with Alzheimer’s disease (AD). Free copper levels in the serum and brain of AD patients are notably elevated, leading to reduced antioxidant defenses and mitochondrial dysfunction. Moreover, free copper accumulation triggers a specific form of cell death, namely copper-dependent cell death (cuproptosis). This article aimed to review the correlation between copper dysregulation and the pathogenesis of AD, along with the primary pathways regulating copper homoeostasis and copper-induced death in AD. Additionally, the efficacy and safety of natural and synthetic agents, including copper chelators, lipid peroxidation inhibitors, and antioxidants, were examined. These treatments can restore copper equilibrium and prevent copper-induced cell death in AD cases. Another aim of this review was to highlight the significance of copper dysregulation and promote the development of pharmaceutical interventions to address it.

## 1 Introduction

Alzheimer’s disease (AD) is a neurodegenerative condition characterized by cognitive memory impairment and is the primary form of senile dementia, representing 60–70% of all dementia cases (Gao et al., 2022; Huang, 2023). As the population ages, the incidence of Alzheimer’s has steadily increased, resulting in substantial societal and financial burdens (Huang, 2023). Earlier studies estimate that around 152 million individuals worldwide will develop AD by 2050, emphasizing the urgent need for effective drug therapies (Zhang et al., 2021). Currently, drugs can only alleviate the symptoms of AD and do not cease or alter its progression. Therefore, it is critical to discover efficient remedies for this condition that pose a notable obstacle for humanity.

Alzheimer’s disease (AD) is characterized by the accumulation of amyloid-beta (Aβ) plaques and tau neuronal tangles in the brain (van der Kant et al., 2020; Gao et al., 2022). However, the etiology and pathogenesis of AD have remained elusive due to the intricate pathological changes (Kaur D. et al., 2021). Two clinical trials targeting Aβ plaques as a therapeutic intervention for AD have not yielded positive outcomes, suggesting that Aβ plaques may not be the optimal target (Salloway et al., 2014). Therefore, the current research is focused on identifying additional pathomechanisms relevant to AD.

In recent decades, an increasing number of studies have linked copper (Cu) dyshomeostasis to the pathogenesis of AD (Squitti et al., 2014; Talwar et al., 2017; Pal et al., 2021a). Cu is a redox-active metal that participates in several metabolic processes in the brain under physiological conditions (Lei et al., 2021). It is either present as protein-bound Cu or non-protein-bound Cu (free Cu) in human tissues, and exerts its effects in the free form (Zhang et al., 2016). Construction of a premature aging model (CuSO4-SIPS) using copper sulfate (CuSO4) demonstrates the role of Cu in age-related functional decline and advancement in age-related diseases (Matos et al., 2015, 2017). Furthermore, higher levels of free Cu have been observed in the serum of patients with AD than in non-AD subjects, with a greater proportion of exchangeable Cu in the brains of patients with AD (Desai and Kaler, 2008; James et al., 2012; Squitti et al., 2014). Furthermore, high consumption of copper sulfate pentahydrate by mice is causally linked to cognitive impairment, suggesting a potential correlation between Cu exposure and AD (Noda et al., 2013; Chen et al., 2022; Zhang et al., 2023).

Cuproptosis is a recent term coined to refer to cell death caused by the aggregation of lipoylated proteins and proteotoxic stress from excessive accumulation of Cu (Tsvetkov et al., 2022). The literature reports a potential correlation between a higher proportion of labile Cu levels and oxidative damage in the brains of patients with AD (James et al., 2012), which is intricately associated with cuproptosis. Therefore, this review focuses on the impact of free Cu and cuproptosis on cognitive functions, as well as potential therapeutic interventions. We believe that a better understanding of the mechanism by which Cu and cuproptosis promote AD development will help in delineating the pathogenesis of the disease and offering insights into effective treatment strategies.

## 2 Copper and cuproptosis

Copper is known to participate in the physiological functioning of the human body (Jomova et al., 2022; Tsvetkov et al., 2022). However, excessive accumulation of free Cu is linked to several pathological conditions, including neurodegenerative disorders, cancer, and cardiovascular diseases (Chen et al., 2022). In Chan et al. (1978) reported that a healthy fibroblast medium containing Cu concentrations above 30 μg/mL induced cell death. Although Cu concentrations in human serum are considerably lower (Barceloux, 1999), Chan et al. (1978) reported that Cu dysregulation contributes to the underlying pathology.

Tsvetkov et al. (2022) reported that excessive Cu^2+^ induces a new form of cell death, which is distinct from all other known apoptotic mechanisms, referred to as cuproptosis. Cuproptosis is mediated by protein lipoylation, and FDX1 serves as the upstream regulator. Thus, both ferredoxin 1 (FDX1) and protein lipoylation mediate cuproptosis. Under physiological conditions, protein lipoylation participates in the tricarboxylic acid (TCA) cycle (Solmonson and DeBerardinis, 2018). Previous studies have shown a correlation between mitochondrial metabolism and susceptibility to cuproptosis. Specifically, cells with an active TCA cycle exhibit increased protein lipoylation, and the lipoyl moiety acts as a Cu binder, further promoting protein lipoylation aggregation, Fe–S cluster protein loss, and heat shock protein (HSP)70 induction, ultimately causing acute protein toxic stress (Tsvetkov et al., 2022). Cuproptosis has been associated with several pathological conditions, including neurodegenerative diseases, Wilson’s disease, cancer, and cardiovascular disease (Chen et al., 2022).

## 3 Copper homeostasis and cuproptosis in the pathogenesis of AD

**FIGURE 1:**
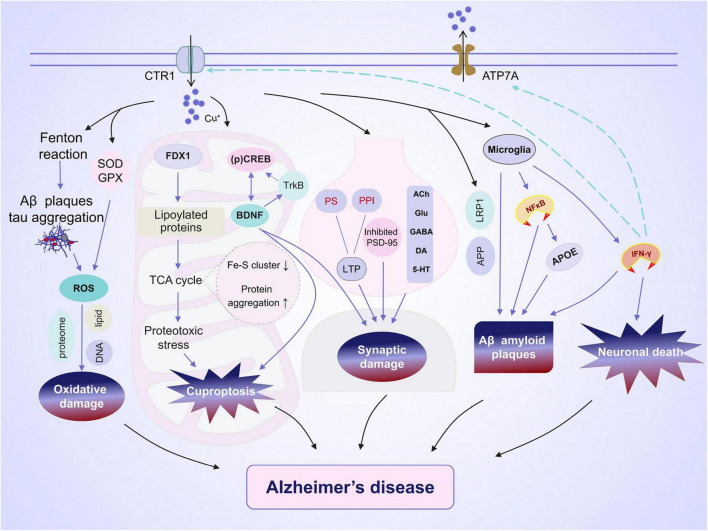
Alzheimer’s disease (AD) and copper ions. Copper is transported into brain cells through membrane copper transporter protein CTR1 and exits through ATP7A. The effect of copper on the development of AD encompasses a varied and intricate array of outcomes. (1) Copper regulates the expression of amyloid precursor protein (APP) and the aggregation of tau, both of which contribute to oxidative damage. (2) Accumulation of copper can result in cuproptosis and reduced activity in the cAMP-response element-binding (CREB) pathway, potentially leading to impaired synaptic plasticity and cognitive abilities. (3) Copper is closely linked to synaptic plasticity via the regulation of long-term potentiation (LTP) and neurotransmission. (4) The excessive accumulation of copper impacts microglial phagocytosis of Aβ and the release of pro-inflammatory cytokines. (5) IFN-γ boosts copper uptake by increasing copper-dependent translocation of ATP7A from Golgi to cytoplasmic vesicles as well as by upregulating the expression level of the copper importer, CTR1. This leads to a significant alteration in copper homeostasis.

Copper has been implicated in the maintenance of normal brain functions, and deviations from appropriate levels, whether insufficient or excessive, can cause several disorders (Madsen and Gitlin, 2007). Excessive Cu accumulation can result in Cu toxicity, leading to apoptosis, astrocytosis, and damage to the hippocampus (Kalita et al., 2018). Therefore, maintaining normal Cu levels is essential for optimal memory and learning. A significant amount of free Cu is present in the serum of patients with cognitive impairment and AD (Squitti et al., 2005, 2017; Rozzini et al., 2018). Moreover, a meta-analysis revealed a positive correlation between serum Cu levels and the risk of AD (Li et al., 2017). Excessive accumulation of Cu could contribute to the development of AD by mediating cuproptosis, oxidative stress, synaptic damage, Aβ plaque deposition, and neuronal death (Figure 1).

### 3.1 Copper causes oxidative stress within the CNS

The central nervous system (CNS) is greatly affected by Cu exposure (Rossi-George and Guo, 2016; Barca et al., 2019). A recent study demonstrated that Cu inhibits the expression of enzymatic antioxidants, namely superoxide dismutase (SOD) and glutathione peroxidase (GPX), in the brain tissue of mice (Zhang et al., 2023), indicating the induction of oxidative damage. In addition, Cu increased the levels of malondialdehyde (MDA), a lipid peroxide, in the hippocampus of rats with cognitive impairment, suggesting lipid damage (Zhang et al., 2023). In another study, Cu exposure elevated the levels of hippocampal nitric oxide and oxidative stress (OS), increased hippocampal tissue lipid peroxidation (LPO) levels, and decreased SOD and catalase (CAT) activities (Lamtai et al., 2020). Furthermore, Cu toxicity induces neuronal death and astrocyte proliferation in the hippocampus through glutamate and oxidative stress pathways, causing impaired memory and learning ability (Kalita et al., 2018). Thus, Cu-induced cognitive impairment is intricately associated with the induction of oxidative damage in the hippocampus (Kalita et al., 2018; Lu et al., 2022).

Copper exists in different oxidation states (Cu^2+^ and Cu^+^) during the Cu cycle, resulting in the dysregulation of Cu steady-states, which is hypothesized to be one of the key mechanisms underlying brain injury (Zhang et al., 2023). Copper can generate reactive free radicals through the Fenton reaction and directly interact with Aβ plaques and amyloid precursor protein (APP), thereby promoting the synthesis and aggregation of Aβ plaques (Kitazawa et al., 2016). Furthermore, Cu can bind to Aβ plaques, thereby directly contributing to the generation of reactive oxygen species (ROS) (Cheignon et al., 2018). The overproduction of ROS can trigger oxidative damage in several biological macromolecules, including the proteome, lipids, and DNA (Wu and Cederbaum, 2003; Tomanek, 2015; Pal et al., 2021b). In contrast, sequestering Cu from Aβ peptides can hinder its accumulation and maximize Aβ degradation (Cherny et al., 2001; Behbehani et al., 2012), inhibit hydroxyl radical (OH) production and oxidative damage, and ultimately decrease cell death (Chen et al., 2022).

The effects of Cu on memory function and oxidative stress in rats are gender-dependent, with marginally greater effects observed in female rats (Lamtai et al., 2020). This could be because estrogen enhances Cu retention, rendering females more susceptible to its neurotoxic effects (Amtage et al., 2014). However, further investigation is warranted to elucidate potential gender differences in the neurocognitive consequences of Cu.

Numerous studies have suggested that Cu-induced oxidative stress and lipid peroxide production could be used as an intervention target to prevent AD.

### 3.2 Copper increases the risk of AD

Copper can interact with several pathogenic factors, including Aβ plaques and tau (Chen et al., 2022). In addition, it promotes the toxic accumulation of Aβ plaques in the brain (Kitazawa et al., 2016), thereby increasing the risk of AD.

The formation and accumulation of Aβ are central to AD pathogenesis. These originate from Aβ precursor protein (AβPP), which deposits in neuronal plaques, wherein AβPP regulates Aβ synthesis (Selkoe, 2001; An et al., 2022). Cu binds to the Aβ protein to form a stable complex (Atrián-Blasco et al., 2019) that generates ROS (Atrián-Blasco et al., 2018) and exacerbates neuronal damage (An et al., 2022). Thus, Cu-promoted Aβ neurotoxicity could be responsible for the pathogenesis of AD.

A study reported that Cu governs the conversion of AβPP into Aβ (Becaria et al., 2006), thereby contributing to the neurodegeneration in AD. In addition, Kitazawa et al. (2016) stated that the Cu–Aβ complex downregulates the expression of lipoprotein receptor-related protein 1 (LRP1), eventually limiting the clearance of neurotoxic Aβ plaques and causing their accumulation in the brain. In contrast, Cu chelators upregulate the expression of ADAM 10 through the melatonin receptor (MT1/2) and its associated downstream signaling pathways, causing marked improvements in cognitive performance in AβPP/PS1 Tg mice (Wang et al., 2018c). Moreover, Cu can phosphorylate tau proteins, causing their aggregation and enhancing the formation of plaques and pathological tangles in the brain (Kitazawa et al., 2009; Du et al., 2014; Voss et al., 2014). Cognitive impairment in AD is attributed to synaptic damage caused by p-tau, both structurally and functionally (Polydoro et al., 2009; Tai et al., 2012).

### 3.3 Effects of copper on the CREB signaling pathway

The accumulation of Cu in the brain is known to trigger cuproptosis and reduce the phosphorylation of cAMP-response element-binding protein (CREB), as well as downregulate the expression of brain-derived neurotrophic factor (BDNF) and its receptor, tropomyosin receptor kinase B (TrkB) (Zhang et al., 2023). In turn, these changes lead to both structural and functional alterations in neurons, which subsequently impairs synaptic plasticity and cognitive abilities.

Furthermore, Cu ions can directly bind to fatty acylated proteins in the mitochondrial tricarboxylic acid (TCA) cycle, thereby stimulating abnormal oligomerization of fatty acylated proteins. This results in a loss of Fe-S cluster proteins, causing proteotoxic stress and, eventually, cuproptosis in neuronal cells. Several studies have demonstrated that neuronal loss can cause cognitive impairment (Hwang et al., 2006; Do Val-da Silva et al., 2016; Esquerda-Canals et al., 2019). The key pathological processes leading to cuproptosis are FDX1-mediated protein lipoacylation and Cu reduction reactions.

Copper accumulation-induced oxidative damage can inhibit CREB phosphorylation and impair CREB-mediated neuronal excitability, which is vital for memory formation (Lisman et al., 2018; Sharma and Singh, 2020; Lu et al., 2022). Phosphorylation of CREB promotes neuronal survival and neutrophin-induced differentiation (Bonni et al., 1995). BDNF is a neurotrophic factor widely distributed throughout the brain, which largely regulates synaptic maturation at morphological, molecular, and functional levels (Nieto et al., 2013). Intracellular signaling, stimulated by both BDNF and its receptor TrkB, contributes to neuronal survival, morphogenesis, and plasticity (Numakawa et al., 2010). In addition, CREB is known to mediate the neurotrophic and neuroprotective impacts of BDNF (Fujino et al., 2009; Wang et al., 2018a), BDNF promotes the phosphorylation of CREB by activating TrkB (Pizzorusso et al., 2000), and CREB phosphorylation promotes the transcription of the BDNF gene (Wang et al., 2018a). This sequence of events is critical for the initiation and preservation of synaptic functions. In addition, increasing the expression of CREB is known to prevent cuproptosis (Zhang et al., 2023).

Because excessive accumulation of Cu in the hippocampus can severely impair cognitive function through CREB in AD, it is essential to maintain Cu homeostasis in the hippocampus. Similarly, activating the CREB pathway and inhibiting neuronal cell death in the AD brain tissue could potentially serve as novel strategies to develop drugs against AD or intervene in it either by delaying or halting its progression.

### 3.4 Effects of copper on microglia

Several studies have identified that pro-inflammatory pathways in microglia are associated with abnormal Cu homeostasis and AD (Zheng et al., 2010; Wang et al., 2018b). Although microglia can phagocytize Aβ or Aβ-antibody complexes (Das et al., 2003; Ennerfelt et al., 2022; Jäntti et al., 2022), an excessive accumulation of Cu ions could hinder phagocytosis. This could be attributed to the effect of Cu on the LDL receptor-related protein 1 (LRP1), which is responsible for transporting Aβ protein (Singh et al., 2013). Increased expression of LRP has been demonstrated to control Aβ accumulation and neuroinflammation in AD (Deane et al., 2008). Therefore, reduced microglial phagocytosis of Aβ and a decrease in LRP1 expression increase the risk of AD following Cu exposure.

Interferon (IFN)-γ is the only cytokine in the IFN family (Conlon et al., 2019) that elicits and facilitates inflammatory responses (Langer et al., 2019; Karki et al., 2021; Yang et al., 2022) and induces cellular death (Kano et al., 1997; Tirotta et al., 2012). IFN-γ transcription and production are significantly increased in reactive microglia and astrocytes surrounding the Aβ deposits in the cerebral cortical region of the transgenic AD mouse model (Abbas et al., 2002). Thus, IFN-γ could exert an inflammatory effect on the formation of amyloid plaques and activation of microglia and astrocytes. IFN-γ induces significant changes in copper homeostasis by increasing copper-dependent transport of ATP7A from the Golgi apparatus to cytosolic vesicles, enhancing copper uptake and raising expression levels of the CTR1 copper importer (Zheng et al., 2010). The finding infer that pro-inflammatory conditions associated with AD substantially alter microglial copper transport, which may account for the fluctuations in copper homeostasis in AD patients (Zheng et al., 2010).

The activation of nuclear factor (NF)-κB in microglia is associated with oxidative stress, inflammatory response, and apoptosis (Liu et al., 2020, 2021; Tastan et al., 2021). Copper facilitates the activation of microglia and ensues neurotoxicity via the phosphorylation and translocation of NF-κB p65. Paired helical filament (PHF) tau and advanced glycation end products in AD generate oxygen radicals that activate transcription via NF-κB, increase Aβ protein precursors, and release Aβ peptides (Yan et al., 1995).

The promoter activity of apolipoprotein E (APOE), which is intricately linked to the development of AD, is dependent on NF-κB (Du et al., 2005). APOE impedes microglial response, blocks Aβ clearance, hastens Aβ aggregation, affects tau pathology and tau-mediated neurodegeneration, and compromises synaptic integrity and plasticity, thereby contributing to the development of AD (Yamazaki et al., 2019).

### 3.5 Effects of copper on nerve synapses

Synaptic damage is the primary cause of cognitive impairment in AD (Polydoro et al., 2009; Tai et al., 2012). A study reported that Cu, an essential element, could exert a biphasic impact on the initiation of neurotransmission to uphold sufficient synaptic function (Opazo et al., 2014). Physiologically, Cu is essential for normal synaptic functions by improving neurotransmission via modification of the arrangement of neuronal proteins, such as by facilitating the accumulation of postsynaptic density protein (PSD) 95 protein (Opazo et al., 2014). Excessive Cu exposure is known to impact both pre- and post-synaptic regulatory mechanisms (Zhang et al., 2023). Copper sulfate (5 mg/kg) has been reported to substantially enhance the population spike (PS) amplitude elicited by hippocampal Schaffer collateral fiber stimulation and repress long-term potentiation (LTP) and paired-pulse index (PPI) in the hippocampal CA1 zone. Conversely, higher concentrations (10 mg/kg and 15 mg/kg) of copper sulfate fail to exert any significant effects on PS amplitude, LTP, or PPI inception (Jand et al., 2018). Another study reported that Cu administration caused a concentration-dependent increase in the total Cu content in the hippocampus. However, hippocampal-free Cu only increased after the administration of lower concentrations (0.2 mg/Kg) of Cu(OAc)2 and decreased after the administration of higher concentrations (2 mg/Kg and 20 mg/Kg) (Zhang et al., 2016). This could be attributed to higher concentrations of Cu that stimulate responses in Cu-binding proteins (Zhang et al., 2016).

Several studies have reported that Cu impedes long-term potentiation and reduces synaptic plasticity (Doreulee et al., 1997; Leiva et al., 2003; Goldschmith et al., 2005). Goldschmith administered high doses of copper (dissolved in water) (8–10 mg/day) to rats and observed inhibition of LTP onset, with a significant reduction in synapse sensitivity and facilitation (Goldschmith et al., 2005). Similarly, Leiva et al. (2003, 2009) investigated the effect of long-term copper sulfate administration (1 mg/kg) on hippocampal LTP, and the findings align with the previously mentioned results. Overall, these findings indicate that copper exerts an effect on synaptic function (Gaier et al., 2013).

In addition, Cu-induced dysregulation of acetylcholine (ACh), glutamate, γ-aminobutyric acid (GABA), and other synaptic transmitters, is strongly associated with cognitive abnormalities and behavioral changes in AD (Pal et al., 2013; Pavandi et al., 2014; Zhang et al., 2016; Kaur S. et al., 2021). Copper is released into the synaptic cleft, where it inhibits excitatory neurotransmission by blocking glutamate receptors (Opazo et al., 2014). However, its excessive accumulation significantly decreases the levels of dopamine, 5-hydroxytryptamine, and GABA and increases those of glutamate (Kaur S. et al., 2021). Glutamate contributes to neurological disorders by binding with glutamate receptors (Traynelis et al., 2010). An excessive amount of glutamate has been demonstrated to induce oxidative stress and activate caspase-3 and glial fibrillary acidic protein (GFAP) (Kalita et al., 2018). The overabundance of extracellular Glu and subsequent excessive activation of ionic Glu receptors eventually cause neuronal cell death (Lamtai et al., 2020). In addition, excessive accumulation of Cu decreases the serum acetylcholinesterase activity and causes neuronal degeneration (Pal et al., 2013). A recent study concluded that exposure to copper downregulates the expression of serotonin (5-HT), dopamine (DA), and GABA in the hippocampus of mice (Zhang et al., 2023).

## 4 Potential therapeutic avenues

### 4.1 Copper chelators

Heavy metals are known to initiate free radical-mediated chain reactions, causing oxidative deterioration of biomolecules, lipid peroxidation, protein oxidation, and oxidation of nucleic acids (Flora et al., 2013). Free metals significantly contribute to the pathomechanisms of AD. Because patients with AD often experience disruptions in metal homeostasis and OS within their brains (Sestito et al., 2019), metal chelators could be used as a potential therapeutic strategy to prevent excessive free Cu from contributing to redox reactions. Metal chelators have been demonstrated to significantly reduce the generation of ROS and hydroxyl radicals (Csire et al., 2020).

Clioquinol (CQ) is a mild metal chelating agent in the body for iron, Cu and zinc (Cahoon, 2009). For example, a 9-week CQ treatment inhibited and could have reversed the accumulation of Aβ deposits in APP 2,576 transgenic animals. Furthermore *in vitro* experiments demonstrated that CQ blocked the interaction of Cu^2+^ and Zn^2+^ within brain Aβ plaques, thus reversing metal ion-induced aggregation (Cherny et al., 2001). However, several studies have reported that CQ does not exhibit a significant advantage over alternative chelators in effectively treating AD (Adlard et al., 2008; Sun et al., 2022). Furthermore, clinical trials of CQ for AD have been unsuccessful (Ho et al., 2022).

5,7-Dichloro-2-(dimethylamino)methyl-8-hydroxyquinoline (PBT2) is a second-generation derivative of 8-OH quinoline (Lannfelt et al., 2008) that has been developed as an ion carrier (Bush, 2008; Cahoon, 2009). It has demonstrated satisfactory efficacy in phase IIa clinical trials (Bush, 2008). However, the results of existing clinical trials are questionable including phase IIa trials. For example, there exists a bias toward reporting outcomes of clinical trials of therapeutic Cu chelators as positive and beneficial for patients (Drew, 2017). In addition, a recent study demonstrated that PBT2 could not chelate Cu(II) from Aβ(1-42) than CQ and B2 Q. Therefore, it can be hypothesized that PBT2 has a poor anti-AD effect (Summers et al., 2022). Further studies are required to completely and accurately assess the protective properties of PBT2 in AD.

Tetrathiomolybdate (TTM) is a kind of chelating agent that can inhibit Cu absorption, has a rapid onset of action, and does not cause neurological deterioration (Ejaz et al., 2020). Although TTM is toxic following injection into the hippocampus, the formation of metal ion/TTM complexes with Cu^2+^ reduces the toxicity (Armstrong et al., 2001). TTM can form a high-affinity triple complex with Cu and albumin, chelating Cu in the bloodstream (Yu et al., 2006). However, ammonium preparations have been considered highly unstable for routine use (Ejaz et al., 2020). Moreover, TTM is not effective when used as a single agent (Yu et al., 2006) and its clinical applicability remains limited (Weiss et al., 2017).

Bis-choline TTM (WTX101) is an orally available Cu protein-binding molecule that targets Cu in hepatocytes and reduces plasma non-ceruloplasmin-bound copper (NCC) by forming a triple complex with albumin and enhances biliary Cu excretion, and is more stable than TTM (Weiss et al., 2017).

Weiss et al. (2017) conducted a Phase II study to evaluate the efficacy and safety of WTX101 in the initial or early treatment of patients with Wilson’s disease (WD). The results suggested that low-dose WTX101 could be a promising novel treatment for WD (Weiss et al., 2017). Molybdenum accumulates in the major organs of Sprague–Dawley rats following repeated oral administration of bis-choline TTM. Therefore, it is hypothesized that prolonged exposure could result in adverse pathophysiological cellular functions (Foster et al., 2022).

Sun et al. (2022) designed specific Cu chelators [tetradentate monoquinolines (TDMQs)] and investigated changes in protein profiles expressed in 5xFAD mice following oral treatment with TDMQ20. The results implied that TDMQ20, a Cu chelator, functions on the cholinergic system and mediates Cu homeostasis in the brains of patients with AD and inhibits the deleterious oxidative stress catalyzed by Cu–Aβ complexes, thereby improving cognitive and behavioral performance in AD rats (Sun et al., 2022). In another study, Zhao et al. (2021) demonstrated that TDMQ20 reduced memory impairment in a mouse model of AD. However, there exists a dearth of extensive clinical trials assessing the effects of TDMQ20 in human subjects.

In addition to potential side effects and instability, numerous objective reasons restrict the use of Cu chelators (Table 1). Certain chelators for Cu are hydrophilic and require small hydrophobic molecules to cross the blood–brain barrier (BBB) (Kenche and Barnham, 2011). However, nanoparticle delivery systems could be promising and intriguing methods for metal chelation in AD treatment (Bonda et al., 2012). This novel technology has been used in multiple studies to treat different illnesses (Hernando et al., 2016; Xiao et al., 2022; Guadarrama-Escobar et al., 2023). Furthermore, Cu is known to maintain proper physiological functions (Opazo et al., 2014; Jomova et al., 2022; Tsvetkov et al., 2022). However, chelating agents involve the risk of depleting the body’s essential Cu levels (Andersen, 2004), adding to the limitation on chelator usage. Thus, it is imperative to explore safer Cu chelators.

**TABLE 1:** Potential therapies targeting copper in AD.

Category	Drug	Limitations
Copper chelators	CQ	Unsuccessful clinical trials
	PTB2	Clinical trials could be biased
	TTM	Unstable efficacy
	WTX101	Molybdenum accumulation
	TDMQ20	No large-scale clinical trials have been conducted to assess safety
Antioxidants	Lazaroids	Failure to sustain cytoprotection during advanced stages of cellular injury
	Curcumin	Induces pharmacokinetic changes
	ECGG	Affects drug effects
	Quercetin	Affects drug effects
	RES	Affects drug effects

### 4.2 Antioxidants

The generation of ROS and accumulation of lipid peroxides have been known to contribute to cuproptosis. It could be feasible to employ antioxidants to impede cuproptosis to treat AD.

In Hall (1992) discovered a range of efficient lipid peroxidation inhibitors known as “lazaroids,” which can alleviate such peroxidation in the brain tissue and impede the degeneration of motor nerve fibers, contributing to the treatment of AD. However, the inability of the Lazaroid compound to sustain cytoprotective effects during the advanced stages of cellular injury could be ascribed to its restricted clinical effectiveness (Huang et al., 2001). Another study demonstrated that a combination of multi-antioxidant nutritional supplements improved the memory and cognitive functions of adults living in the community (Chan et al., 2010).

Natural polyphenols, including resveratrol, epigallocatechin gallate, and curcumin, are potential therapeutic agents in treating oxidative stress, reducing extracellular amyloid deposition, and managing AD (Jayasena et al., 2013).

Curcumin is extracted from turmeric and possesses therapeutic properties, including anti-inflammatory, neuroprotective, neurotoxic metal chelating, anti-amyloidogenic, and antioxidative effects on mitochondria and DNA (Lakey-Beitia et al., 2017; Khayatan et al., 2023; Maghool et al., 2023). This natural extract also modifies microglia activity and inhibits acetylcholinesterase, making it an effective treatment for AD (Tang and Taghibiglou, 2017). However, its potential therapeutic value may be limited by its low bioavailability (Tang and Taghibiglou, 2017). There may be hope for curcumin-based treatments for AD in the future, provided that the issue of poor bioavailability can be effectively addressed (Tang and Taghibiglou, 2017).

A study demonstrated that green tea extract, epigallocatechin-3-gallate (EGCG) can reduce the toxicity of metal-free Aβ and metal-Aβ plaques and exhibit a distinctive anti-amyloidogenic reaction to metal-aβ plaques (Hyung et al., 2013). However, several studies have demonstrated that high concentrations of EGCG can cause liver toxicity (Wang et al., 2015a,b).

Quercetin belongs to the flavonol class of flavonoids; it is one of the most effective plant antioxidants (Brüll et al., 2015). Quercetin protects neural cells by attenuating neuroinflammation (Bournival et al., 2012) and inhibiting Aβ aggregation and tau phosphorylation (Khan et al., 2019), anti-oxidative, anti-lipid peroxidative, and acetylcholinesterase inhibitory activities (Rishitha and Muthuraman, 2018). However, its limited penetration via the BBB restricts its efficacy against neurodegenerative diseases (Khan et al., 2019).

Resveratrol (RES) is an effective antioxidant, scavenger of ROS, and a metal chelator (Olas and Wachowicz, 2005). A previous study demonstrated that RES treatment protected high-fat diet (HFD)-induced insulin resistance (IR) rats from diet-induced IR and elevated the expression of sirtuin 1 (SIRT1) and sirtuin 3 (SIRT3), mitochondrial DNA, and mitochondrial biogenesis (Haohao et al., 2015). In addition, RES enhances mitochondrial antioxidant enzyme activity, thereby reducing oxidative stress (Haohao et al., 2015).

Naturally occurring polyphenols possess considerable therapeutic potential due to their intrinsic antioxidative and chelating properties. In addition, certain polyphenols can potentially cross the BBB through chemical modification of their structure (Grabska-Kobyłecka et al., 2023). However, potential interactions between antioxidants and other medications should be considered as patients with AD could have additional underlying conditions requiring long-term treatment (Table 1). For instance, resveratrol can influence the effectiveness of other medications by inhibiting intestinal enzymes such as CYP3A4 or P-glycoprotein, especially in drugs with higher first-pass effects, such as specific calcium channel blockers, sildenafil, midazolam, and nefazodone (Detampel et al., 2012). In addition, quercetin and EGCG have been reported to display a similar effect (Choi and Burm, 2009; Choi et al., 2011; Tang et al., 2023). Curcumin can cause changes in pharmacokinetics during its simultaneous use with pharmacological agents such as cardiovascular drugs, antidepressants, anticoagulants, antibiotics, chemotherapeutic agents, and antihistamines (Bahramsoltani et al., 2017).

### 4.3 Other therapeutic targets

It has been established that Cu can activate the NF-κB signaling pathway, causing increased production of several inflammatory factors (Liu et al., 2020, 2021; Tastan et al., 2021) and APOE activity activation (Du et al., 2005), ultimately driving AD progression. Therefore, the application of NF-κB inhibitors could hinder the activity of NF-κB-induced APOE, consequently obstructing the progression of AD.

Reports have stated that p53 can inhibit glucose uptake and glycolysis, promoting a metabolic shift in the TCA cycle and oxidative phosphorylation and contributing to Cu toxicity (Li et al., 2023; Xiong et al., 2023). In addition, p53 regulates the biogenesis of Fe-S clusters and glutathione (GSH) (Ho et al., 2022; Xiong et al., 2023), thereby mitigating or enhancing Cu toxicity (Xiong et al., 2023)—two processes crucial for cuproptosis. Thus, p53-related therapeutic targets could be associated with cuproptosis, although its detailed mechanism of regulating Cu toxicity requires further research.

Alternatively, because Cu significantly contributes to the complex pathological mechanisms underlying AD, treating patients with multiple heterozygous compounds acting simultaneously on different biological targets could be a promising approach (Dias and Viegas, 2014). For instance, a clinical trial demonstrated that administering a nutritional formulation containing folic acid, α-tocopherol, B12, S-adenosylmethionine, N-acetylcysteine, and acetyl-L-carnitine to patients with AD for over a year effectively delayed AD progression (Remington et al., 2016).

## 5 Conclusion and future perspectives

The majority of clinical studies based on the mainstream hypothesis of AD pathogenesis have not yielded significantly positive outcomes. Therefore, it is essential to unravel the complex pathophysiological mechanisms underlying AD and discover and develop novel AD therapeutic targets and drugs. Despite the potential of Cu-chelating agents, lipid peroxidation inhibitors, antioxidants, and other molecules to prevent homeostatic disorders and cuproptosis, we need to identify drugs that can effectively cross the BBB with minimal systemic side effects. In this regard, nanomedicine delivery systems present a promising avenue for AD therapy. However, it is crucial to emphasize the possibility of drug–drug interactions in patients simultaneously taking multiple medications.

Major challenges for forthcoming studies entail comprehensively identifying the roles of copper in AD pathogenesis and potential druggable targets. Our team has been dedicated to investigating methods to improve cognitive capabilities for an extended period. Our next goal is to develop a copper-targeted therapy for the treatment of AD. We aimed to provide a scientific basis for the clinical development of novel treatment approaches for AD, which is a promising and challenging process.

In conclusion, our review of the mechanisms of oxidative stress, cuproptosis death, amyloid deposition, neuronal death, and synaptic damage in AD caused by Cu dysregulation demonstrated that Cu and cuproptosis are promising therapeutic avenues for the future treatment of AD through the development of safe and effective Cu-chelating drugs.

## Author contributions

XL: Investigation, Writing – original draft. XC: Investigation, Writing – review & editing. XG: Formal analysis, Supervision, Writing – review & editing.
